# Canine CD117-Specific Antibodies with Diverse Binding Properties Isolated from a Phage Display Library Using Cell-Based Biopanning

**DOI:** 10.3390/antib8010015

**Published:** 2019-02-12

**Authors:** Mohamed A. Alfaleh, Neetika Arora, Michael Yeh, Christopher J. de Bakker, Christopher B. Howard, Philip Macpherson, Rachel E. Allavena, Xiaoli Chen, Linda Harkness, Stephen M. Mahler, Martina L. Jones

**Affiliations:** 1 Australian Institute for Bioengineering and Nanotechnology (AIBN), The University of Queensland, Brisbane, QLD 4072, Australia; mohamed.alfaleh@uqconnect.edu.au (M.A.F.); narora86@hotmail.com (N.A.); uqmyeh@gmail.com (M.Y.); chris.debakker@servatus.com.au (C.J.d.B.); c.howard2@uq.edu.au (C.B.H.); x.chen8@uq.edu.au (X.C.); l.harkness@uq.edu.au (L.H.); s.mahler@eng.uq.edu.au (S.M.M.); 2 Faculty of Pharmacy, King Abdulaziz University, Jeddah 21589, Saudi Arabia; 3 Vaccines and Immunotherapy Unit, King Fahd Medical Research Center (KFMRC), King Abdulaziz University, Jeddah 21589, Saudi Arabia; 4 Australian Research Council Training Centre for Biopharmaceutical Innovation, The University of Queensland, Brisbane, QLD 4072, Australia; 5 Centre for Advanced Imaging, The University of Queensland, Brisbane, QLD 4072, Australia; 6 School of Veterinary Science, The University of Queensland, Gatton, QLD 4343, Australia; philip.macpherson@uq.net.au (P.M.); r.allavena@uq.edu.au (R.E.A.)

**Keywords:** Affinity selection, cancer, canine, CD117, c-Kit, dog, immunohistochemistry, membrane proteins, monoclonal antibody, phage display, sphere culture, transfection, veterinary oncology, whole cell panning

## Abstract

CD117 (c-Kit) is a tyrosine kinase receptor that is overexpressed in multiple dog tumors. There is 100% homology between the juxtamembrane domain of human and canine CD117, and many cancer-causing mutations occur in this region in both species. Thus, CD117 is an important target for cancer treatment in dogs and for comparative oncology studies. Currently, there is no monoclonal antibody (mAb) specifically designed to target the exposed region of canine CD117, although there exist some with species cross-reactivity. We panned a naïve phage display library to isolate antibodies against recombinant CD117 on whole cells. Several mAbs were isolated and were shown to bind recombinant canine CD117 at low- to sub-nanomolar affinity. Additionally, binding to native canine CD117 was confirmed by immunohistochemistry and by flow cytometry. Competitive binding assays also identified mAbs that competed with the CD117 receptor-specific ligand, the stem cell factor (SCF). These results show the ability of our cell-based biopanning strategy to isolate a panel of antibodies that have varied characteristics when used in different binding assays. These in vitro/ex vivo assessments suggest that some of the isolated mAbs might be promising candidates for targeting overexpressed CD117 in canine cancers for different useful applications.

## 1. Introduction

Membrane proteins represent 20% to 30% of all proteins in most living entities and have a major role in transportation and signal transduction [1,2]. They account for 44% of human drug targets, which make them the biggest group of drug targets and one of the most attractive targets for drug discovery [3,4]. Monoclonal antibodies (mAbs) against membrane proteins are highly desirable as research reagents and also as diagnostic and therapeutic agents. MAbs are created specific to a cell surface antigen, and a large amount of research and development is focused on the utility of mAbs as standalone drugs, antibody–drug conjugates, or antibody-targeted, drug-loaded nanoparticles for cancer therapy [5,6,7,8,9]. The desirable properties of an mAb depend upon its final application, whether that be for diagnosis or for therapy. For example, immunohistochemistry is commonly used for testing formalin-fixed tissue sections, which requires mAbs that can bind denatured or linear epitopes. Meanwhile, therapy and in vivo diagnosis require mAbs that can bind natively folded target antigens on the cell surface.

**Figure 1:**
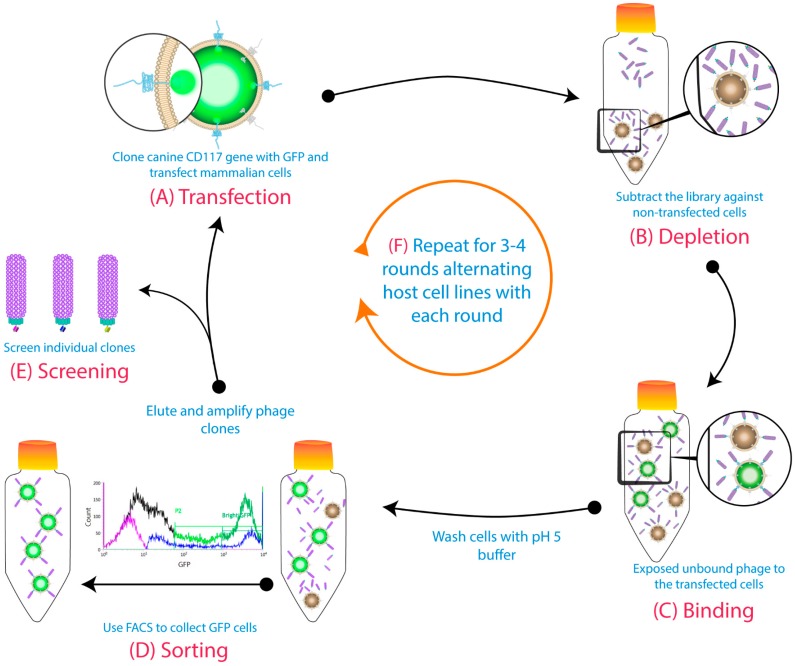
Cell-based biopanning protocol using cell sorting and alternative host cell lines: (**A**) The gene for the target membrane protein was cloned in-frame with Green Fluorescent Protein (GFP) to be expressed on the cell surface. (**B**) The phage library was subtracted on untransfected cells. (**C**) Then, the free phages were panned against transiently transfected cells. (**D**) A pH 5 wash step was conducted before GFP-positive cells were collected using fluorescence-activated cell sorting (FACS). The binders from the sorted cells were eluted with a low pH buffer, then amplified for the subsequent rounds. (**E**) Individual clones were screened at the end of biopanning campaigns. (**F**) The host cell line was alternated each round (first round: Chinese hamster ovary (CHO) cells; second round: Human embryonic kidney 293 cells (HEK293), etc.). Figure shows methodology described by Jones et al. (2016) [18].

Phage display of antibody libraries is an in vitro method for the isolation of novel mAbs [10,11]. Traditionally, immobilized purified antigens are used during the biopanning of phage libraries. However, membrane proteins are difficult to purify in their native conformation, and immobilization onto a surface may mask or alter important epitopes. Biopanning of purified protein may isolate antibodies that are unable to bind the native membrane proteins. Alternatively, cell-based biopanning eliminates the need for antigen purification and immobilization and preserves the natural hydrophobic–hydrophilic interactions, which are necessary for maintaining conformation and stability [12,13]. This allows isolation of biologically relevant antibodies that recognize naturally exposed epitopes [14,15,16]. Unfortunately, there are significant challenges when biopanning phage libraries on whole cells, including the low density of target antigens, the high background of irrelevant antigens, and the intrinsic ability of phages to nonspecifically adhere to the cells. Numerous strategies for selecting membrane protein-specific antibodies using a phage display in conjunction with cell-based biopanning have been employed to address these limitations [17]. In our lab, we optimized a cell-based biopanning method that uses transient expression of the target membrane protein fused to Green Fluorescent Protein (GFP) on the surface of mammalian cells, combined with the use of fluorescence-activated cell sorting to isolate antibodies against membrane proteins, specifically human CD83, canine CD117, and bat CD11b [18]. This biopanning method is outlined in Figure 1. Here, we further describe the isolation and characterization of seven unique mAbs, isolated using this method, that bind to canine CD117 and that may be useful for a variety of purposes due to their differing characteristics.

Canine CD117 (c-Kit), which is a tyrosine kinase receptor [19,20,21], was chosen for antibody discovery because there are no available mAbs that specifically target the extracellular domain of the dog receptor. CD117 receptors are usually present on the cell surface as monomers, and the processes of dimerization and autophosphorylation are initiated directly after binding to a specific hematopoietic growth factor known as stem cell factor (SCF) [22,23]. As with other tyrosine kinase receptors, CD117 is tightly regulated, and any deregulation caused by mutations, overexpression, chromosomal translocation, or autocrine activation can promote constitutive receptor activation, even in the absence of SCF [23,24]. In dogs, CD117 overexpression has been detected in different kinds of dog cancers, such as mast cells tumors (MCTs) and gastrointestinal stromal tumors (GISTs) [25,26,27,28,29,30,31].

In both humans and dogs, higher expression levels of CD117 have been reported in cancerous cells compared to normal cells and have been associated with a poor prognosis [32,33,34,35,36]. Thus, the dog is an appropriate model for studying human CD117-induced tumors, because the overall amino acid sequence of the canine CD117 is 88% homologous with human CD117, and the juxtamembrane domain (exon 11), where similar mutations are observed in both species, is 100% homologous [37,38,39]. Additionally, a significant number of dogs are diagnosed with cancer every year, and their naturally and spontaneously occurring malignancies are valuable tools for comparative oncology, especially for exploring human cancer, since genetic alterations, histological appearance, and response to traditional therapies for canine and human tumors have confirmed that both share similar characteristics [38,40,41,42,43,44]. Comparative oncology studies aim to help researchers achieve a better understanding of cancer biology and to help translate new cancer treatments from dogs to humans and vice versa.

Here, we characterized binding specificity and affinity of seven unique mAbs, isolated from a naïve antibody phage library using cell-based biopanning. The antibodies were found to differ in their binding properties toward native, recombinant, and denatured CD117, and in their ability to inhibit binding of SCF. Thus, the isolated mAbs are promising candidates for targeting overexpressed canine CD117 either for research, diagnosis, or therapy: However, further studies associated with their biological function(s) need to be conducted.

## 2. Materials and Methods

### 2.1. Cloning Protocol for Canine CD117-GFP and SCF

The sequence of canine CD117 and SCF was synthesized by GeneArt (Thermo Fisher Scientific, Melbourne, Australia). The CD117 sequence included a secretion signal and extracellular and transmembrane domains (NCBI Reference: NP_001003181; residues 1 to 545). This was cloned in-frame with GFP into the mammalian expression vector pEGFP-N1 (TaKaRa, Mountain View, CA, USA, discontinued), using restriction sites NheI and BamHI to create the vector pEGFP-N1-CD117-GFP. The canine SCF sequence included a secretion signal, the soluble part of the extracellular domain (NCBI Reference: NP_001012753.1; residues 1 to 191), and a 6× His-tag. This was cloned into the mammalian expression vector pcDNA3.1 (+) (Thermo Fisher Scientific), using restriction sites HindIII and XhoI to create the vector pcDNA3.1 (+)-canine-SCF. DNA for transfection was prepared using a PureLink HiPure Plasmid Maxiprep kit (Thermo Fisher Scientific). A control plasmid, pEGFP-N1-CD83-GFP, containing CD83 in place of CD117, was also prepared as described previously [18].

### 2.2. Constructing a pcDNA 3.1 (+)-ECD-CD117-hFc Plasmid for Producing Recombinant Canine CD117-ECD-Human-Fc Fused Protein

The canine CD117 ECD forward primer ATTAA**AAGCTT**CCACCATGAGAGGCGCAAGAGGCG with a HindIII restriction site (bold) and the canine CD117 ECD reverse primer TAT**ACCGGT**GGGGGTGAACAGGGTGTGGGGATG with an AgeI restriction site (bold) were used to amplify the CD117 ECD from a Geneart synthesized CD117 template. Underlined primer regions represent the CD117-ECD specific sequence. The PCR product was inserted into a pcDNA 3.1 (+) mammalian expression vector (Invitrogen) already containing a human hFc insert via HindIII and AgeI-compatible BspEI restriction sites. A positive clone was confirmed by sequencing.

### 2.3. Biopanning

Whole-cell biopanning of transiently expressed canine CD117-GFP fusion protein has been previously described [18]. Briefly, a naïve human phage library obtained from lymphocytes of 50 healthy blood donors using IgG- and IgM-targeted primers with a calculated diversity of 5 × 10^9^ [18] was used. This library, displaying scFv fragments, was depleted against untransfected cells, and then the unbound phages were panned against transiently transfected cells. A pH 5 wash step was conducted to remove loosely bound phages, followed by fluorescence-activated cell sorting of GFP-positive cells. The binders from the sorted cells were eluted with a pH 2.3 elution buffer. These binders were then amplified in XL1-Blue cells, and the phages were rescued to be used for the subsequent rounds, where the host cell line for transient transfection of CD117-GFP was alternated between CHO and human embryonic kidney 293 (HEK293) cells. Individual clones from the last round were isolated for analysis and sequencing.

### 2.4. Reformatting of Phage Clones

Seven unique phage clones were reformatted to whole human IgG1 using a reported ligation-free method [45] into mammalian expression vectors, and then were expressed in CHO cells as described below.

### 2.5. Protein Expression in CHO Cells

CHO-S cells (Thermo Fisher Scientific) were cultured in CD-CHO with 8-mM GlutaMAX (Thermo Fisher Scientific) at 37 °C and 7.5% CO_2_, with shaking at 130 rpm. On the day of transfection, cells were prepared at ~3.0 × 10^6^ cells/mL at greater than 98% viability. Cell surface expression of the CD117-GFP fusion protein was performed as described previously [18], and cells were used 2 days post-transfection. For soluble expression of CD117-hFc, reformatted hIgGs, and canine SCF, transient transfection was prepared as follows: 600 µg total of reformatted IgG plasmids (equal amounts of heavy and light chain plasmids) or pcDNA 3.1 (+)-ECD-CD117-hFc DNA or pcDNA3.1 (+)-canine-SCF were mixed with 15 mL of OptiPRO SFM (Thermo Fisher Scientific) and incubated at room temperature (RT) for 30–60 s. Concurrently, 2.4 mL of PEI-Pro (Polyplus-transfection, New York, NY, USA) was mixed with 30 mL of OptiPRO SFM and incubated at RT for 30–60 s. The PEI-Pro complex was then added to the DNA complex by gentle pipetting and incubated statically at room temperature for 15 min. The DNA complex was then added to 200 mL of CHO culture in a shake flask and mixed by gentle swirling. The culture was incubated at 37 °C and 7.5% CO_2_, with shaking at 130 rpm for 4 to 6 h, after which the culture was diluted 1:2 (*v*/*v*) with CD-CHO supplemented with final concentrations of EfficientFeed A and B (Thermo Fisher Scientific) 7.5% each (*v*/*v*), 4% Anti-Clumping Agent (Thermo Fisher Scientific), and 8 mM of GlutaMAX (Thermo Fisher Scientific). The culture was incubated at 32 °C, 7.5% CO_2_, 130 rpm for 10 to 14 days and then supernatant-harvested by centrifugation and filtered using a 0.22-µm filter.

### 2.6. Protein Purification

The expressed products were purified using Protein A affinity chromatography, utilizing the Fc domain for IgGs and CD117-hFc. After loading the culture supernatant, the Protein A column (GE Healthcare) was washed with phosphate-buffered saline (PBS), and the reformatted anti-canine CD117 IgG mAbs or ECD-CD117-hFc were eluted using 0.1 M of glycine pH 3 and neutralized with 1 M of Tris-HCl (pH 9.0). His-tagged SCF was purified using immobilized metal affinity chromatography with a 5-mL HisTrap excel column (GE Healthcare, Sydney, Australia). The column was equilibrated in 20-mM sodium phosphate pH 7.4 + 500 mM NaCl. The harvested supernatant was loaded onto the column and then washed with equilibration buffer containing 20 mM of imidazole. SCF was then eluted with 20 mM of sodium phosphate pH 7.4 + 500 mM NaCl + 500 mM imidazole. Eluted proteins were desalted into PBS using a HiPrep 26/10 column (GE Healthcare) and then filtered using a 0.22-µm filter. The concentration of eluted protein was determined by using absorbance at 280 nm using a Nanodrop 1000 spectrophotometer (Thermo Fisher Scientific) and the calculated extinction coefficient.

The purity and the molecular mass of the protein of interest were determined by using SDS-PAGE. In brief, Protein™ 4%–12% Bis-Tris, SDS-Polyacrylamide gel (Invitrogen), was used with Nu PAGE^®^ LDS sample buffer (4×) as a loading buffer and Nu PAGE^®^ sample reducing agent (10×) as a denaturing buffer (Thermo Fisher Scientific), or water in the case where sample reducing was not required. The gel was run in 1% MES buffer for 35 min at 200 V, followed by staining with SimplyBlue SafeStain (life technologies) for 1 h at RT. SeeBlue plus 2 standard molecular mass marker (Invitrogen) was used to estimate the molecular mass of the purified proteins.

### 2.7. Flow Cytometry Analysis of Anti-CD83 and Anti-CD117 Reformatted IgGs

CHO-s cells transfected with pEGFPN1-CD117-GFP or pEGFPN1-CD83-GFP plasmids were used for flow cytometry analysis two days post-transfection. The cells were washed in 10 mL of PBS and resuspended in 10 mL of 2% Milk-PBS (MPBS) and kept on rotation at RT for 1 h. Concurrently, 4-μg/mL purified IgG mAbs prepared in 500 µL of 2% MPBS were incubated, rotating at RT for 30 min. Then, the blocked mAbs were added to 500-µL aliquots of blocked cells for 1 h at 4 °C for both the CD117- and CD83-expressing cells. The incubated cells were centrifuged at 200× *g* for 3 min and then washed twice with 500 µL of PBS. All samples were resuspended in 500 µL of DyLight594-antihuman IgG Fc (Abcam) antibody diluted 1/1000 in 2% MPBS. Samples were incubated for 30 min at 4 °C and washed twice with 500 µL of PBS. Finally, all samples were resuspended in 1 mL of PBS for cytometric analyzing on a Cube8 flow cytometer (Sysmex, Gorlitz, Germany) using a 561-nm laser and a 630/22-nm filter to measure the secondary antibody fluorescence, and a 488-nm laser and a 536/40-nm filter to measure the GFP fluorescence. The data were analyzed using FCS Express Flow version 4 (De Novo Software, Glendale, CA, USA).

### 2.8. Enzyme-Linked Immunosorbent Assay (ELISA)

The sensitivity of the anti-canine CD117 mAbs toward recombinant soluble CD117-hFc protein was measured using ELISA. In brief, a Nunc maxisorb plate was coated with 200 µL per well of recombinant CD83 (negative control) or canine CD117-hFc at 1 µg/mL in PBS overnight at 4 °C. The wells were then washed three times with PBS-T and blocked with 350 µL per well of 2% MPBS for 1 h at RT. Simultaneously, in a separate 96-well round-bottom plate, 4 µg/mL of purified IgGs was blocked and serially diluted 1 in 3 with 2% MPBS in 200 µL total volume, and incubated at RT for 1 h. Then, the ELISA plate was decanted and loaded with 200 µL/well of the blocked serially diluted IgGs and incubated at RT, followed by 3× washes with PBS-T. This was followed by the addition of 200 µL of αHu Kappa (AFF)-PEROX (The Binding Site, Birmingham, UK) (1/5000 dilution in 2% MPBS) or Anti-Lambda Light chain antibody (HRP) (Abcam, Cambridge, UK) (1/3000 dilution in 2% MPBS) was added into each well and further incubated at RT for 1 h, then washed 3× with PBS-T. Finally, the plate was washed at least three times with PBS-T before adding 100 µL of 3,3′,5,5′ tetramethylbenzidine (TMB) (Sigma-Aldrich, Sydney, Australia) to initiate the reaction and was incubated for 5–10 min. The reactions were terminated by adding 100 µL of 2-M H_2_SO_4_ per well, and the absorbance was measured at 450 nm.

Competitive ELISA was conducted to detect the ability of the phage-derived reformatted mAbs to block the canine CD117/SCF interaction. First, the optimized concentration of SCF was determined to be 50 ng/mL for this assay. For the blocking analysis, mAbs were serially diluted 1 in 2, starting at 40 µg/mL in 200-µL 2% MPBS in a separate 96-well plate, and were then incubated at RT for 1 h. Then, a constant concentration of 50 ng/mL of canine SCF was added to all the wells. This mixture was then added to ELISA plates coated with 1 µg/mL canine CD117-hFc and incubated at RT for 1 h, followed by 3× washes with PBS-T. Then, 200 µL anti-His HRP (Miltenyi Biotec, Sydney, Australia) (1/4000 dilution in 2% MPBS) secondary antibody was added into each well and further incubated at RT for 1 h, then washed 3× with PBS-T. Finally, the plates were washed three times with PBS-T before adding 100 µL of 3,3′,5,5′ TMB (Sigma-Aldrich) to start the reaction, and were left for 5–10 min. The reactions were terminated by adding 100 µL of 1-M H_2_SO_4_ per well, and the absorbance was measured at 450 nm.

### 2.9. Affinity and Kinetics Analysis Using Surface Plasmon Resonance

The interaction kinetics and affinities of the IgG1 mAbs (D12-κ, H1-κ, C11-κ, G4-κ, C3-λ, E8-λ, and E7-λ) with the recombinant canine CD117-hFc were measured using SPR performed on a Biacore T200 instrument (GE Healthcare). The assay design utilized immobilized CD117-hFc as the ligand and antibodies in solution as the analytes. Prior to amine coupling of CD117-hFc to the CM5 chip, a pH scouting experiment determined that the optimal immobilization buffer was 10 mM of sodium acetate, pH 5. The Amine Coupling Kit (GE Healthcare) was used to immobilize CD117-hFc to the CM5 chip on flow cells 4 and 2, aiming for 100 resonance units (RU) and 3600 RU, respectively. The CM5 chip surface was activated by injection of 70 μL of a 1:1 mixture of 0.4-M *N*-ethyl-*N*’-(dimethylaminopropyl) carbodiimide (EDC) and 0.1-M *N*-hydroxysuccinimide (NHS) at a flow rate of 30 µL/min. Then, CD117-hFc was injected at 10 µg/mL in 10 mM of sodium acetate buffer, pH 5. This was followed by an injection of 70 µL of 1-M ethanolamine (pH 8.0) to block the remaining activated groups on the surface of the CM5 chip. Flow cells 1 and 3 were activated and blocked with ethanolamine and were used as the reference cells. Single-cycle kinetics methodology was then used to evaluate binding of the mAbs to the CD117-hFc. For each mAb, 5 serially diluted concentrations (0.34, 1.02, 3.06, 9.18, 27.54 nM) in HBS-EP+ buffer (GE Healthcare) were injected (low concentration to high) over the immobilized canine CD117 for 180 sec at a flow rate ranging between 30 and 100 µL/min, and were then left to dissociate for 600 s before regeneration of the binding surface with 10 mM of glycine, pH 2.0, for 30 s at 10 µL/min. Reference subtractions of flow cells (2-1) and (4-3) were incorporated in the analysis, as were blank and buffer-only sample runs. Sensorgrams were analyzed using BiaEvaluation software Version 1.0 (GE Healthcare), and a 1:1 binding model was used to fit kinetic variables.

### 2.10. Immunohistochemistry (IHC)

Formalin-fixed, paraffin-embedded (FFPE) 4-micron sections of canine mast cell tumors were prepared by routine methods on superfrost slides. Tumors were previously confirmed as high-grade mast cell tumors by a board-certified veterinary pathologist [46]. The sections were dewaxed in xylene, rehydrated in graded alcohols, and deionized in distilled water. Heat-induced antigen retrieval was conducted by immersing the slides in 0.01-M Citric Acid-Based Buffer, pH 6.0 (Vector Laboratories, Burlingame, CA, USA) and incubating them for three periods of 5 min in a microwave (650 W). Then the slides were allowed to cool down for 25 min at RT. Endogenous peroxidase activity was quenched by incubating the slides with methanol containing 1% hydrogen peroxide for 40 min at RT. Then the slides were washed using TBS-T (Abcam) for 5 min and blocked by 5% normal goat serum in TBS-T for 30 min RT. Then an avidin/biotin blocking step was conducted using an Abcam endogenous avidin/biotin blocking kit. All the reformatted antibodies, and a commercial mouse antihuman CD117 (Clone YB5.B8, BD Biosciences, Sydney, Australia) known to cross-react with canine CD117 [47], were diluted to 30 µg/mL in TBS, pH 7.4 (Abcam), while for the negative control section, TBS buffer alone substituted the primary antibody. Then the slides were incubated overnight at 4 °C in a covered humidity chamber. The following day, the slides were washed in TBS-T, incubated with biotinylated secondary goat Anti-Human IgG Fc or Anti-Mouse IgG (H&L), F(ab)’ fragment (Life Technologies), and diluted 1/500 in TBS for 30 min at RT. After washing in TBS-T, the slides were incubated with ABC substrate solution (Vector Laboratories) for 30 min. Then the slides were washed in TBS-T and incubated with DAB solution until the desired color developed (8 min), followed by distilled water rinsing. The slides were counterstained with hematoxylin for 45 s and dehydrated, fixed, and mounted with xylene mounting medium.

### 2.11. Adherent and Non-Adherent Spheres Culture

The canine hemangiosarcoma cell line DD1 (Kerafast, EMN009) was prepared and supplied by Dr. Jaime Modiano (Masonic Cancer Center, University of Minnesota, Minneapolis, MN, USA). The cells were cultured as an adherent monolayer as described previously [48], with one modification, where accutase solution (Sigma-Aldrich) was used for monolayer detachment during passaging. Cells were maintained at 37 °C with a 7.5% CO_2_ atmosphere and were re-fed every 2 to 3 days. Non-adherent hemangiosphere formation of DD1 was achieved by growing the early passages of the adherent cells under serum-free conditions that favored non-adherent sphere formation for at least three weeks, as described previously [49]. Spheres were maintained at 37 °C with a 7.5% CO_2_ atmosphere in an untreated 100-mm^2^ Petri dish with 3 × 10^6^ cells replacing the medium every 3 to 4 days. The media were supplemented with 10 µg/mL basic fibroblast growth factor (bFGF) (PeproTech, Rocky Hill, NJ, USA) every 24 h, since fibroblast growth factor activity is diminished within 24 h at 37 °C [50]. Spheres were enzymatically dissociated using accutase solution into single-cell suspensions once per week for routine culture maintenance.

### 2.12. Flow Cytometry of Canine Hemangiospheres Cell Line DD1

Each of the isolated anti-canine CD117 mAbs (D12-κ, H1-κ, C11-κ, G4-κ, C3-λ, E8-λ, and E7-λ) and the commercial mouse antihuman CD117 (Clone YB5.B8, BD Biosciences) were used as primary antibodies. Goat Anti-Human IgG Fc (DyLight^®^ 594) (Abcam) and Goat Anti-Mouse IgG H&L DyLight^®^ 594 (Abcam) were used as secondary antibodies. Detection of CD117 was performed as follows: Single-cell suspensions from non-adherent hemangiospheres, maintained for three weeks, were washed twice with 500 µL of FACS staining buffer (PBS with 2% fetal bovine serum (FBS)). The cells were incubated on ice for 20 min with 1000 µL of 2% BSA/FACS staining buffer to block nonspecific binding. Cells were treated with 500 µL of 30-µg/mL primary mAbs for 45 min on ice, then washed twice with FACS staining buffer. Cells were then incubated with 500 µL of secondary antibody for 20 min on ice, followed by 3× washes in 500 µL of FACS staining buffer, and were then resuspended in PBS. Fifty thousand events per sample were collected for flow cytometric analysis on a Cube8 flow cytometer (Sysmex) using a 561-nm laser and a 630/22-nm filter. The data were analyzed using FCS Express Flow version 4 (De Novo Software).

## 3. Results

### 3.1. Biopanning

Analysis of the phage pools after each round of biopanning and individual clones after four rounds of biopanning have been previously reported [18]. Alternating the transfected cell line in each round of cell-based panning reduced the isolation of irrelevant binders and increased the yield of scFv-phage specific to canine CD117. Sorting cells after phage binding allowed us to isolate binders from only the antigen-positive cells, which are the cells that highly express canine CD117 as a fusion protein with GFP, and therefore the yield of binders for specific antigen was increased (Table 1). Sorting also allowed the removal of dead cells and cellular debris, ensuring that the isolated phages were eluted from the cell surface and not from intracellular or degraded proteins.

Sequence analysis of clones that bind specifically to recombinant canine CD117 transfected cells revealed seven unique clones, four of which were identified as kappa (κ) isotypes (D12, C11, H1, and G4) and three as lambda (λ) isotypes (C3, E8, and E7). Of these clones, three were found to be similar (D12-κ, C11-κ, H1-κ), with differences observed only in their light-chain CDR1 and CDR3 regions.

### 3.2. Expression of Proteins

**Figure 2:**
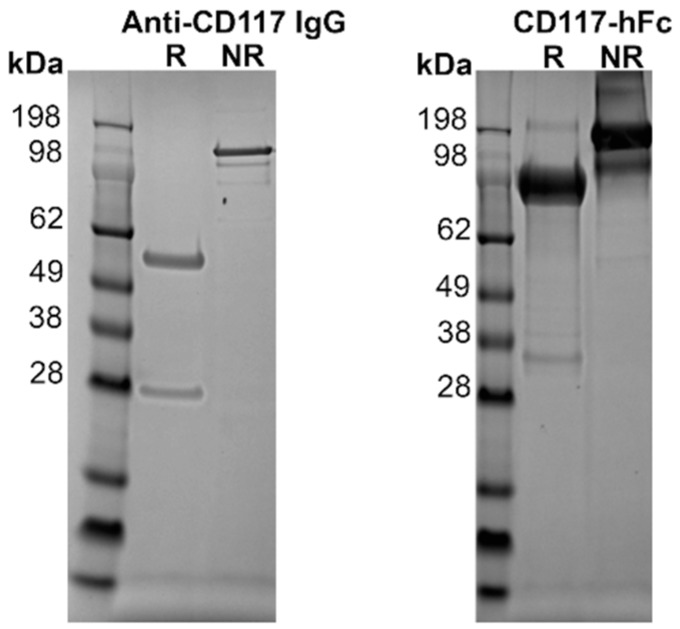
On the left, purified recombinant reformatted fully human IgG and recombinant CD117-hFc fusion protein in 4%–12% SDS-PAGE. Size standard is SeeBlue Plus 2 molecular mass marker (Invitrogen). The stained gel of H1-κ as a representative purified human anti-canine CD117 IgG antibody showed the light-chain at ~25 kDa and the heavy chain at ~50 kDa under reducing (R) conditions, and the full IgG at ~145 kDa under nonreducing (NR) conditions. On the right, purified Fc fused canine CD117 extracellular domain (ECD) showing the presence of the monomeric form of the CD117-hFc at ~84.2 kDa under reducing conditions, and a dimer of ~168.4 kDa under nonreducing conditions.

The seven unique clones were reformatted into fully human IgG mAbs in a manner described previously [45] in order to improve the avidity and to allow testing against various sources of canine CD117. The seven mAbs were expressed in Chinese hamster ovary (CHO) cells and purified by Protein A affinity chromatography. Analysis using polyacrylamide gel electrophoresis with sodium dodecyl sulphate (SDS-PAGE) showed the non-reduced sample formed a complete antibody at ~145 kDa, while the reduced sample showed the heavy and light chains at ~50 kDa and ~25 kDa, respectively (Figure 2).

The extracellular domain (ECD) of canine CD117 fused to human Fc (hFc) was expressed recombinantly for binding studies with the mAbs. Transfected CHO cells yielded 14 mg of CD117-hFc protein from a culture volume of 200 mL. The recombinant protein was purified with Protein A affinity chromatography. SDS-PAGE analysis in nonreducing conditions showed a protein band at a size consistent with a dimer formed via disulfide linkage in the hFc domain. This disulfide bond was reduced to form monomers under reducing conditions (Figure 2).

For the expression of canine SCF-His-tag, transfected CHO cells yielded 40 mg of SCF-His-tag protein from 200 mL of culture. The recombinant protein was purified by immobilized metal affinity chromatography with a HisTrap excel column (GE Healthcare). Analysis by SDS-PAGE showed the expected molecular mass to be ~19.3 kDa (data not shown).

### 3.3. Flow Cytometry Analysis of Antibody Binding to Recombinant Cell Surface Canine CD117

**Figure 3:**
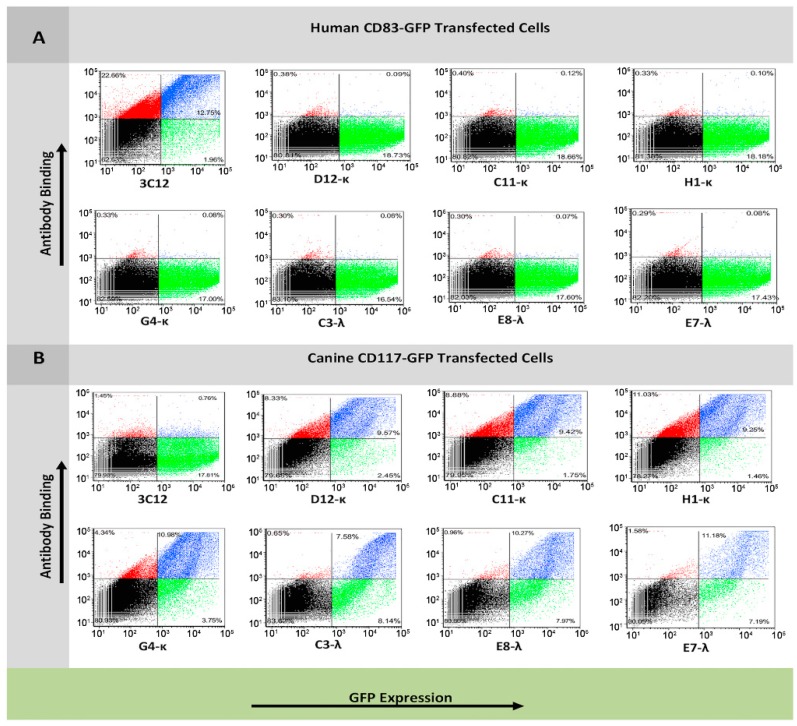
Binding specificity of anti-canine CD117 IgG1 reformatted clones isolated by cell-based biopanning for CD83-GFP- and CD117-GFP-expressing CHO cells by flow cytometry. Cells were transfected with either CD83-GFP fusion plasmid (**A**) or CD117-GFP fusion plasmid (**B**). Cells were incubated with either 3C12 (anti-CD83 monoclonal antibody (mAb)) or one of the seven anti-CD117 mAbs. Antibody binding was detected using Dylight 594 antihuman IgG secondary antibody. Secondary antibody fluorescence (*y* axis) was measured using a 561-nm laser and a 630/22-nm filter. GFP fluorescence (*x* axis) was measured using a 488-nm laser and a 536/40-nm filter.

To examine whether the reformatted mAbs bind specifically to CD117-ECD and not to GFP or other CHO cell antigens, the seven mAbs were tested using flow cytometry for binding to CHO cells transfected with either pEGFPN1-mem-CD117-GFP or pEGFPN1-memCD83-GFP as a negative control. Each of the seven mAbs bound to CD117-GFP transfected cells proportional to the level of GFP expression (blue population), but did not bind to CD83-GFP cells, indicating that the binding was specific to CD117-ECD and not to GFP or other CHO antigens (Figure 3).

### 3.4. Binding and Competition Assays Using ELISA

**Figure 4:**
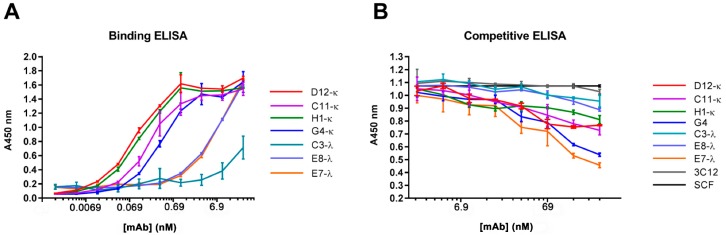
ELISA for anti-canine CD117 fully reformatted IgG1 mAbs. (**A**) The ELISA plate was coated with recombinant canine CD117-hFc, then probed with the seven anti-canine CD117 IgG1 mAbs. Bound mAbs were detected using horse radish peroxidase (HRP) conjugated anti-κ and anti-λ secondary antibodies. (**B**) The ELISA plate was coated with recombinant canine CD117-hFc and then probed with different concentrations of anti-canine CD117 mAbs pre-mixed with 50-ng/mL recombinant canine stem cell factor (SCF)-His. The presence of SCF was detected using anti-His HRP secondary antibodies. Error bars represent standard deviations.

All seven mAbs were able to bind to the recombinant CD117 (Figure 4A), with mAbs D12-κ, C11-κ, H1-κ, and G4-κ showing greater sensitivity than C3-λ, E8-λ, and E7-λ mAbs, although the different specificities of the anti-kappa and anti-lambda secondary antibodies may have been contributing to this difference. In addition, ELISA showed that all the mAbs were able to compete with the CD117-specific ligand SCF, with different levels of inhibitory effect. G4-κ and E7-λ demonstrated the strongest inhibitory effect, and C3-λ and E8-λ showed the weakest. D12-κ, C11-κ, and H1-κ demonstrated moderate competing abilities with SCF to CD117 (Figure 4B).

### 3.5. Binding Assessment of Canine-Specific Anti-CD117 mAbs to Recombinant Canine CD117 Target Using Surface Plasmon Resonance (SPR)

**Figure 5:**
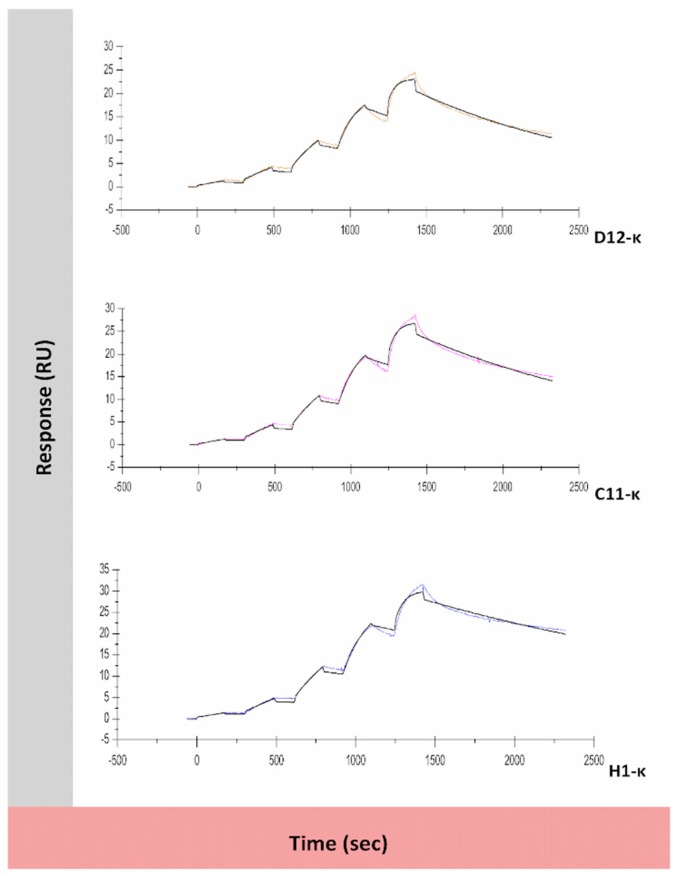
Surface plasmon resonance (SPR) sensorgrams for the kinetic interaction between anti-canine CD117 IgG1s (analyte) and the canine CD117-hFc (ligand). The CM5 sensorchip was prepared with low-level immobilization, and single-cycle kinetics were performed at a high flow rate of 100 µL/min, allowing a 1:1 binding model to be fit to the data. A 1:1 binding model (black line) was fit to the sensorgrams using BiaEvaluation software, and the calculated kinetic and equilibrium constants were recorded in Table 2.

SPR conditions were set up to allow a 1:1 binding model to be used on the bivalent ligand and bivalent analyte, these conditions being a low level of ligand (CD117) immobilization (90.2 RU) and a high flow rate (100 µL/min). Under these conditions, mAbs D12-κ, C11-κ, and H1-κ were able to bind, and kinetic data and binding affinities were obtained (Figure 5 and Table 2). The experimental *R_max_* (ranging from 27%–37% of the theoretical *R_max_*), and the low Chi^2^ values confirmed the reliability of the data: However, there is still the possibility that some bivalent interactions were occurring despite the assay conditions and were contributing to the apparent high affinity. No binding was evident for mAbs G4-κ, E8-λ, and E7-λ at this low level of immobilized ligand, and a higher level was needed (3640.5 RU) to detect binding (see Figure S1). With this higher immobilization level, a 1:1 binding model was not valid, so kinetic constants could not be determined. Meanwhile, C3-λ showed no binding even at high ligand immobilization. Overall, D12-κ, C11-κ, and H1-κ demonstrated high affinity binding toward CD117-hFc in SPR experiments, while the remaining mAbs did not appear to show any significant interaction.

### 3.6. Anti-Canine CD117 mAbs Binding to Native Canine CD117

**Figure 6:**
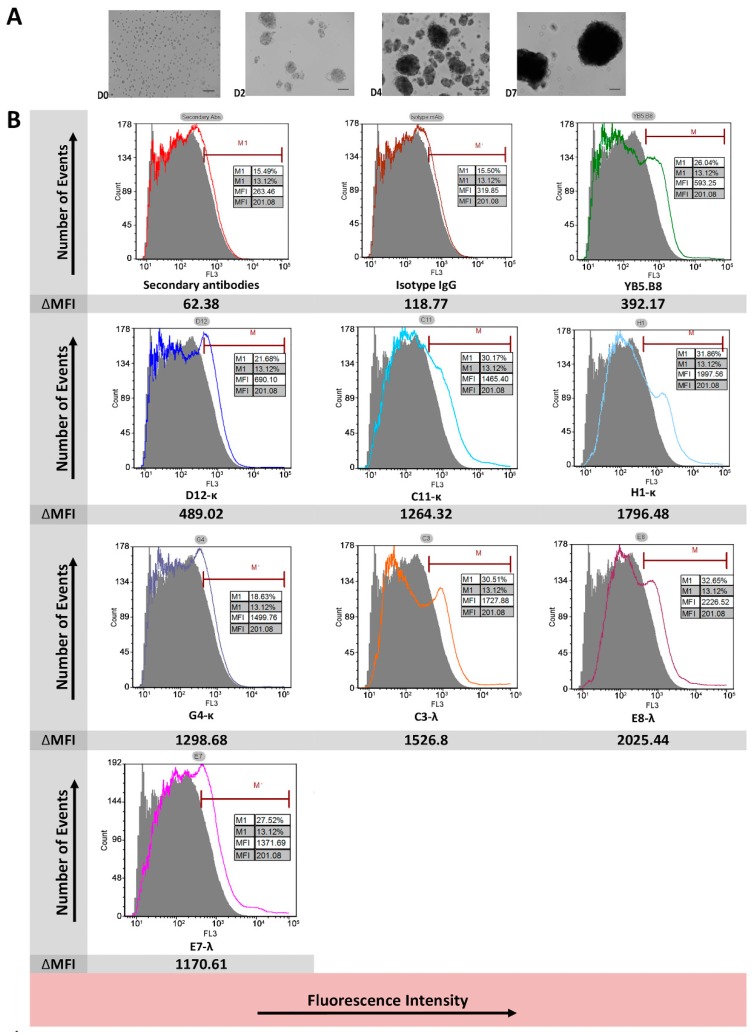
Native canine hemangiosarcoma cells (DD1) expressing CD117 receptors. (**A**) Phase images of canine hemangiosarcoma cells (DD1). Bar = 100 μm. Cells were cultured in an untreated Petri dish plate under non-adherent spheroid-forming conditions. The propagation of the cells was recorded at day 0 (D0), D2, D4, and D7. (**B**) Testing of phage-derived anti-canine CD117 reformatted antibodies against DD1 cells using flow cytometry. Bound mAbs were detected using Dylight 594 antihuman IgG and antimouse IgG secondary antibodies. Fluorescence was measured using a 561-nm laser and a 630/22-nm filter. The solid lines represent the positive staining of the anti-CD117 mAbs. Control (cells alone) is indicated by the grey shaded region. ∆MFI (arithmetic mean fluorescence intensity) represents the change in MFI from the control cells to the antibody-treated cells.

Canine hemangiosarcoma cell line (DD1) [47,51] and formalin fixed paraffin-embedded (FFPE) canine mast cell tumor tissue sections were used as a natural source for canine CD117 in order to assess the reactivity of our canine-specific anti-CD117 mAbs. The expression of CD117 from the canine hemangiosarcoma cell line was achieved when grown under sphere-forming conditions for at least two weeks (2 passages) (Figure 6A) [49]. We tested all the phage-derived, reformatted anti-CD117 mAbs on three-week cultures of hemangiospheres using an antihuman CD117 that cross-reacts with canine CD117 [47] as a positive control (clone: YB5.B8). All the isolated mAbs and YB5.B8 were able to bind to the DD1 cell line (Figure 6B), although at varying levels. YB5.B8, D12-κ, and G4-κ only showed a shift in fluorescence for a small portion of cells, while the other mAbs shifted all the cells, indicating that the former mAbs were weaker and only bound cells with a high level of CD117 expression. All binding mAbs caused the appearance of an additional peak, indicating that the population of cells was heterogeneous with respect to the level of CD117 expression.

**Figure 7:**
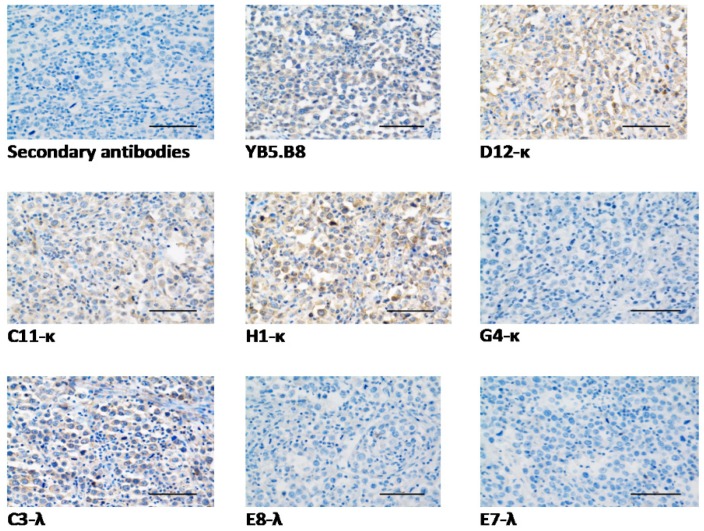
Immunohistochemistry staining of CD117 in high-grade canine CD117 mast cell tumor tissue sections using reformatted anti-canine CD117 mAbs. Bar = 50 μm. Positive mAb binding was detected by diffuse cytoplasmic CD117 protein immunostaining of canine neoplastic mast cells.

Immunohistological staining of FFPE canine mast cell tumor tissue sections confirmed the ability of D12-κ, C11-κ, H1-κ, and C3-λ to target canine CD117 protein expressed in canine mast cell tumor tissue (Figure 7), demonstrating a similar staining pattern to the positive control YB5.B8. The control tissue section, G4-κ, E8-λ, and E7-λ showed no staining.

## 4. Discussion

Canine CD117 is a promising biopharmaceutical target for dog cancer treatment. This hypothesis was confirmed in studies demonstrating that anti-CD117 tyrosine kinase inhibitors (TKIs) suppressed proliferation and stabilized canine MCTs [52,53,54,55]. The canine-specific TKI Palladia (toceranib phosphate) (Zoetis, USA) [56] has been approved for canine MCT treatment: However, it is associated with many severe side effects [52,57]. In case of resistance, mAbs against CD117 offer an alternative to TKIs, potentially with less toxic side effects. With this in mind, we utilized a novel whole cell biopanning approach using a naïve phage library and isolated a panel of seven unique mAbs (Figure 1) [18]. Characterization of these mAbs showed that their binding properties were quite diverse, despite being isolated during the same biopanning campaign.

It was important to confirm that the isolated mAbs were specific for CD117 and not to another cellular antigen. Thus, specificity was confirmed in two alternative recombinant antigen presentations: (i) Transiently expressed cell surface CD117 fused to GFP, measured using flow cytometry; and (ii) soluble purified CD117 fused to hFc, measured using ELISA and SPR. In each of these assays, the κ-mAbs showed stronger binding toward the recombinant canine CD117 in comparison to the λ-mAbs.

The CD117 receptor has three domains: Extracellular (519 aa), transmembrane (23 aa), and intracellular (433 aa) [34]. The extracellular domain is comprised of five immunoglobulin-like domains (D1 to D5) (encoded by exons 1 to 9), wherein the SCF binds to the D1–D3, while D4 and D5 play an essential role in receptor dimerization [27,58,59]. SCF is a CD117-specific ligand [60], and their interaction activates downstream signal transduction pathways, subsequently affecting biological behavior such as proliferation and differentiation of hematopoietic cells, mast cells and interstitial cells of Cajal, and cancer occurrence, growth, metastasis, and recurrence [61,62,63]. Using competitive ELISA, we showed that the mAbs G4-κ and E7-λ were the strongest at interrupting or inhibiting the formation of an SCF/CD117 complex, yet they had lower affinities to CD117 than mAbs D12-κ, C11-κ, and H1-κ, suggesting they bind to different epitopes. It is unclear whether G4-κ and E7-λ compete with SCF for the same site: However, the significance of this assay demonstrates that a cell-based phage display enables the isolation of mAbs binding different epitopes.

The SPR kinetic data separated the mAbs into two distinct groups: (i) D12-κ, C11-κ, and H1-κ, which exhibited high affinity (0.6–0.9 nM) toward immobilized CD117-hFc, and (ii) G4-κ, C3-λ, E7-λ, and E8-λ, which either did not bind in SPR or required a high level of immobilization, making kinetic data unreliable. Upon examination of the six complementarity determining regions (CDRs) of D12-κ, C11-κ, and H1-κ, it was observed that four of the CDRs were identical, namely CDR1, 2, and 3 located on the heavy chain, and CDR2 located on the κ light chain. The sequence similarity between this group of molecules may help to explain why they shared a comparable level of binding affinity (K_D_) and kinetic parameters (k_a_ and k_d_), as determined by SPR. G4-κ, however, had no CDR sequence similarity with the other κ-light chain mAbs and behaved very differently, displaying a lower binding affinity that was comparable to the isolated λ-light chain antibodies. The published K_D_ of antibodies derived from a naive library range between 10^−8^ and 10^−10^ M [64,65,66]: Therefore, the reported K_D_ for the isolated mAbs from the naïve human library fell within the expected range.

Antigen epitopes can be either linear, continuous sequences of amino acids or conformational, where discontinuous amino acids come close together as a result of protein folding into a tertiary structure [67,68,69]. It is important to consider the structure of the targeted epitopes in order to gain insight into the interactions with the antibody. For example, antibodies that are suitable for flow cytometry and ELISA may not work in immunohistochemistry (IHC), because an antibody recognizing a conformation-dependent, discontinuous epitope may not bind to antigens in IHC, where tissue fixation and heat-mediated antigen retrieval during immunohistological staining denatures the conformational epitopes, while the linear epitopes survive [70,71,72]. Thus, our goal was to not only ensure the isolated binders were reactive to native canine CD117, but also to deduce whether their paratopes were continuous or discontinuous epitopes. Accordingly, we tested two native sources of canine CD117: A canine hemangiosarcoma cell line using flow cytometry and FFPE canine mast cell tumor tissue sections using IHC.

Canine hemangiosarcoma cell line DD1 was examined using flow cytometry to confirm that the isolated mAbs were able to bind to the native antigen in its correct three-dimensional structure, which is mandatory for in vivo diagnostic and therapeutic applications. When the canine hemangiosarcoma cells are grown as monolayers, the cells progressively lose expression of CD117 over time to the point where there are fewer than 2%–5% of the cells expressing CD117. However, the proportion significantly increases when the cells are grown in sphere-forming conditions [49]. The expression of the cell markers under non-adherent growth conditions is passage-dependent [49], because spheres dissociating into single cells and then reforming acts as a purification step toward marker enrichment [73]. Consistent with the recombinant canine CD117 protein findings, all the isolated anti-canine mAbs were able to bind DD1 cells when grown as spheroids (Figure 6A). The anti-canine CD117 mAbs showed staining patterns similar to data reported in the literature for DD1 cells [51]. Up to 16.37% of cells bound to the mAbs, which reflected the small percentage of canine hemangiosarcoma cells expressing CD117 (Figure 6B). Flow cytometry data were different between the recombinant transfected cell surface CD117 and the native CD117 from hemangiosarcoma cells. The κ-mAbs showed better reactivity than the λ-mAbs when tested against recombinant cell surface-expressed canine CD117. Meanwhile, the hemangiosarcoma cell line DD1 showed that the λ-mAbs had reactivity similar to C11-κ and H1-κ and appeared to bind better compared to D12-κ and G4-κ. The observed results could reflect the presence of differences in the CD117 isoforms in canine hemangiosarcomas [74] or differences in the receptor density between the transfected cells and the native cells.

IHC staining of MCTs showed significant differences between the mAbs. Only D12-κ, C11-κ, H1-κ, and C3-λ were able to bind to the high-grade canine MCT tissue sections (Figure 7). The staining of the aforementioned mAbs was characterized by faint diffuse cytoplasmic staining of cells, which characterized the high-grade MCTs and is known as pattern III [42,75,76]. G4-κ, E7-λ, and E8-λ were not able to stain the FFPE canine MCT tissue sections. We conclude that the specific epitopes of G4-κ, E7-λ, and E8-λ are conformational and were damaged by the harsh process of the immunohistological tissue-staining protocol, while D12-κ, C11-κ, H1-κ, and C3-λ epitopes are linear and more resilient. The sequence similarities of the mAbs might suggest their tendency to bind to similar epitopes. For instance, D12-κ, C11-κ, and H1-κ have similar CDR sequences, and all of them recognize linear epitopes. This can be investigated and confirmed by performing epitope biopanning and/or mapping experiments.

The reactivity of the different anti-canine CD117 mAbs varied between the assays and the antigen source (Table 3). Therefore, it is recommended that for whole cell-based biopanning campaigns, all of the isolated antibodies should be assessed and analyzed for a range of applications. For example, there were noticeable differences in mAb binding ability among the assays when using native and recombinant sources of canine CD117 (Table 3). This raises a question of whether the SPR results using recombinant, immobilized CD117 truly represented the mAb kinetics within an in vivo system.

The current mAbs may be useful for applications in canine cancer research, diagnosis, and therapy. The phage-derived mAbs were able to bind to different forms of canine CD117 and showed varied binding characteristics in each conducted assay (Table 3). Their ability to be used as therapeutic agents needs to be assessed rigorously using different pre-clinical assays. Moreover, the sequences of their human variable and constant regions must be replaced with corresponding dog sequences to reduce the dog anti-human antibody response [77].

## 5. Conclusions

Here, we isolated mAbs from a naïve antibody library that bind to both recombinant and native forms of the canine CD117 receptor. The reactivity of the individual mAbs differed between different assays and the antigen source (Table 3). Although it was useful to validate the isolated mAbs against soluble recombinant forms of target antigens for specificity and affinity, this did not necessarily represent antibody–antigen interactions in their natural context. These outcomes demonstrated the power of the cell-based biopanning method, which allowed us to isolate a panel of mAbs that could access the native antigen and bind to different epitopes. For instance, biopanning against purified protein immobilized onto a plastic surface may not have isolated binders such as C3-λ, E8-λ, and E7-λ antibodies, which bind weakly in ELISA and SPR but bind well to native CD117 with flow cytometry. We recommend that during whole cell-based biopanning campaigns, all the isolated antibodies should be included in all assessments without exclusion, including those antibodies that seem unpromising during initial analysis. It is essential to validate the isolated mAbs on a soluble recombinant form of the targeted antigen to confirm the specificity and assess the binding affinity and avidity, yet this does not necessarily represent what is naturally occurring between the antibody paratopes and the antigens.

A cell-based phage display is a powerful technique that allowed us to isolate a panel of mAbs with different characteristics. The strength of this technique comes from its ability to isolate antibodies that bind to antigens present in their native or near-native conformation. Moreover, the use of transfected cell lines permits the selection of antibodies to native receptors without the need for expression and purification of the desired protein, accelerating the generation of antibodies that bind the antigen of interest. Therefore, the canine-specific anti-CD117 mAbs that we isolated using a cell-based phage display are clear examples of the potential of this platform for generating binders against membrane proteins.

## Figures and Tables

**Table 1:** Cell-based biopanning selection of canine CD117-specific scFv-phage binders. Phage titer expressed as colony forming units (cfu).

Selection Round	Cell Line	Transfected Cells (cells/mL)	FACS High-GFP Sorted Cells	Phage Input Titer (cfu)	Phage Output Titer (cfu)
Round 1	CHOs	1.3 × 10^6^	~3.0 × 10^6^	1 × 10^13^	1.8 × 10^5^
Round 2	HEK293	1.0 × 10^6^	~3.0 × 10^6^	6.8 × 10^12^	2.1 × 10^5^
Round 3	CHOs	2 × 10^6^	~2.5 × 10^6^	5.2 × 10^12^	2.3 × 10^5^
Round 4	HEK293	1.2× 10^6^	~3.0 × 10^6^	9.2 × 10^10^	8.8 × 10^5^

**Table 2:** Kinetic parameters for the interaction between the anti-CD117 IgG1 and the canine recombinant CD117-hFc immobilized receptor, determined using SPR. A 1:1 binding model was used for the analysis.

mAb	*k_a_* (M^−1^ S^−1^)	*k_d_* (S^−1^)	K_D_ (M)	*R_max_* (RU)	Chi² (RU²)	Theoretical *R_max_* (RU)
D12-κ	8.1 × 10^5^	7.5 × 10^−4^	9.3 × 10^−10^	21.4	0.382	77.9
C11-κ	7 × 10^5^	6.1 × 10^−4^	8.8 × 10^−10^	25.5	0.487	77.9
H1-κ	6.6 × 10^5^	3.9 × 10^−4^	5.9 × 10^−10^	29.0	0.513	77.9

**Table 3:** Summary of anti-canine CD117 mAb characterizations on recombinant versus native canine CD117. Here, 0–4 describes the strength of mAb–CD117 interactions in an ascending order from none (“0”) to very strong (“4”). IHC: Immunohistochemistry.

mAb	Recombinant Canine CD117				Native Canine CD117		
	Flow Cytometry	ELISA	SPR	Ligand Blocking	IHC	Flow Cytometry	Suggested Epitope Type
**D12-κ**	+4	+4	+4	+2	√	+1	Linear
**C11-κ**	+4	+3	+4	+2	√	+3	Linear
**H1-κ**	+4	+4	+4	+2	√	+3	Linear
**G4-κ**	+3	+3	+1	+3	×	+1	Conformational
**C3-λ**	+2	+1	0	+1	√	+3	Linear
**E8-λ**	+2	+2	+1	+1	×	+4	Conformational
**E7-λ**	+2	+2	+1	+3	×	+3	Conformational

## Supplementary Materials

The following are available online at http://www.mdpi.com/2073-4468/8/1/15/s1, Figure S1: SPR sensorgrams for the kinetic interaction between E8-λ, G4-κ, and E7-λ anti-canine CD117 IgG1s (analyte) and the canine CD117-hFc (ligand).
