# Supplementary material for: Canine CD117-Specific Antibodies with Diverse Binding Properties Isolated from a Phage Display Library Using Cell-Based Biopanning
Source: Antibodies (Basel). 2019 Feb 12;8(1):15. doi: 10.3390/antib8010015 (PMC6640692; doi:10.3390/antib8010015)
Supplement: Supplementary file 1 [file antibodies-08-00015-s001.pdf]

Supplementary Information

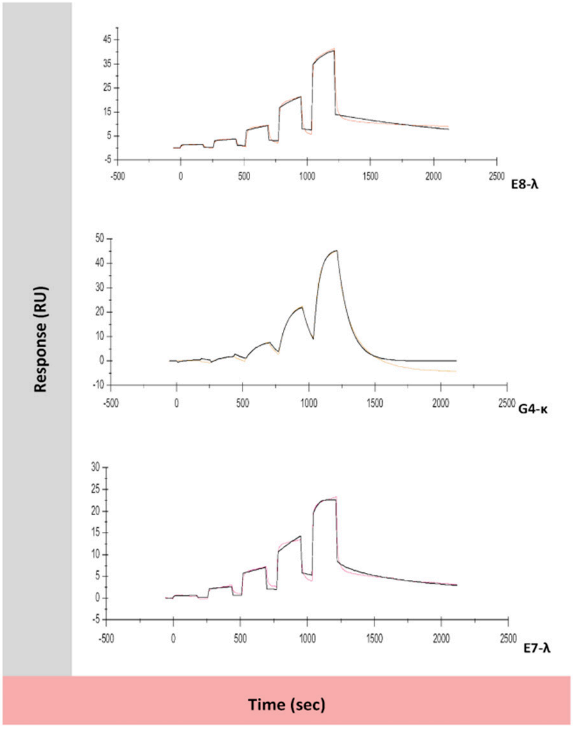

Figure S1. Surface plasmon resonance (SPR) sensorgrams for the kinetic interaction between anti-canine CD117 IgG1s (analyte) and the canine CD117-hFc (ligand). The CM5 sensorchip was prepared with high level immobilisation and single-cycle kinetics was performed at a flow rate of 30 μL/min, allowing a 1:1 binding model to be fit to the data. A 1:1 binding model (Black line) was fitted to the sensorgrams using BiaEvaluation software.
